# High-Volume Plasticizer di(2-Propylheptyl) Phthalate DPHP Induced Ecotoxic Effects in Aquatic and Terrestrial Arthropods

**DOI:** 10.1021/acsenvironau.5c00191

**Published:** 2026-04-05

**Authors:** Anita Jemec Kokalj, Andraž Dolar, Taja Korpar, Aljona Lukjanova, Margit Heinlaan

**Affiliations:** † Biotechnical Faculty, University of Ljubljana, Jamnikarjeva 101, 1000 Ljubljana, Slovenia; ‡ Laboratory of Environmental Toxicology, National Institute of Chemical Physics and Biophysics, Akadeemia tee 23, 12618 Tallinn, Estonia

**Keywords:** DEHP, plastic additives, *Daphnia magna*, *Porcellio scaber*, *Tenebrio molitor*, mealworm, woodlice

## Abstract

With regard to the sustainability of plastics, the role of plastic additives can no longer be ignored or underestimated, as additives may play a more significant role in the biological impact of plastics than anticipated. Plasticizers form the largest group of additives, and phthalates are the best-known plasticizers. To date, several low molecular weight phthalates are restricted/banned due to health and environmental hazards, so various substitute plasticizers, especially high molecular weight phthalates such as di­(2-propylheptyl) phthalate (DPHP), in particular, are increasingly used. However, the hazard data of DPHP, particularly those for environmental organisms, are very limited. The current study investigated the potential long-term impact of substitute plasticizer DPHP in comparison to that of the previously dominant di­(2-ethylhexyl) phthalate (DEHP). As model organisms, aquatic arthropods, such as crustacean *Daphnia magna,* and terrestrial arthropods, such as mealworm *Tenebrio molitor* and woodlice *Porcellio scaber,* were chosen. Exposure to phthalates was carried out via 0.5–1000 mg DPHP/DEHP/kg-spiked sediment, soil, or food, depending on the assay. Solvents were used in spiking, and results are presented in relation to the solvent controls. For *D. magna*, both phthalates induced delayed reproduction in F0 and F1 generations and a smaller parental size at 25 mg/kg. The reduced number of broods (DPHP) and fertility (DEHP) was recorded in the F1 generation. For *T. molitor*, no adverse effects on organism mass, moult, or development were recorded during 8 week exposure at up to 1000 mg/kg food. For *P. scaber*, a 2-week exposure to phthalates did not affect immunity but triggered biochemical responses. DEHP exposure induced potential neurotoxicity (increase in acetylcholinesterase) above 50 mg/kg and detoxification (increase in glutathione S-transferase) above 100 mg/kg, simultaneously reducing metabolic activity. DPHP induced detoxification processes, even at 5 mg/kg. In conclusion, DPHP toxicity induction potential was comparable to that of DEHP, indicating that more hazard data are needed to ensure that DPHP is not a regrettable substitution to DEHP.

## Graphical abstract

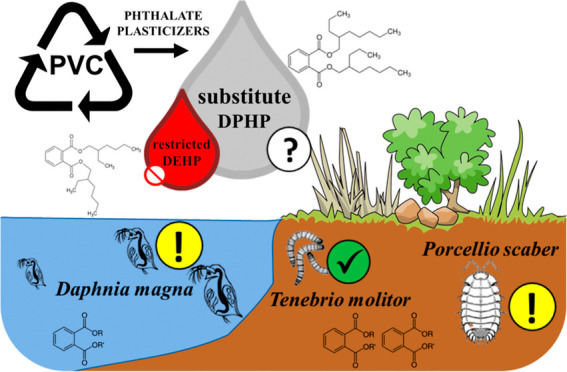

## 1 Introduction

The challenges with the increasing use of plastics go beyond that of differently sized plastic waste in the environment. Only recently has the chemical diversity of plastics started to gain attention as *a* factor affecting plastics’ sustainability,^1^ including, for instance, their toxicity^2,3^ and recyclability.^4^ As a material, plastic is a chemically diverse blend of polymer(s) and additional substances. Over 16,000 chemicals associated with plastic materials and products were documented in a recent PlastChem project,^5^ of which 26% were identified as chemicals of concern, but 66% lacked official hazard classification.^6^ The significant role additives play in the toxicity potential of plastics has been shown with industry effluents,^7^ consumer plastic leachates,^2,3,8,9^ and single-use plastic labware.^10^ However, although causation remains complex in mixed plastic matrices,^9,11^ growing evidence now identifies additive-driven effects. Knowing this is important. There are currently great efforts to negotiate an international, legally binding instrument for plastic pollution. Plastics-associated chemicals, including plasticizers, are mentioned explicitly in the Chair’s Text of UNEP.^12^ Plasticizers, that render plastics flexible, are one of the most important groups of functional additives both in terms of representation^13^ as well as concentration.^12^ Phthalic acid esters ((ortho)­phthalates) are the dominant plasticizers, of which several have been increasingly restricted and/or banned in the European Economic Area (EEA) for more than 20 years because of their toxicity (Table S1). Anticipating the phasing out of the most toxic low molecular weight (<C4) phthalates, the plastic industry has turned to alternatives—potentially less hazardous isomeric, high molecular weight (C7+) phthalate, and nonphthalate plasticizers (e.g., organophosphates and citrates). By now, many of these substitute plasticizers have become emerging contaminants.^14,15^ Low molecular weight (C6) di­(2-ethylhexyl) phthalate (DEHP) has been the dominant phthalate plasticizer; however, due to legislative measures (Table S1), its use in the EEA has dropped by 97% to less than 10,000 tonnes.^16^ The functionality of DEHP in plastics is difficult to replace; thus, it has not been entirely phased out and is mostly restricted (Table S1). The main substitution to DEHP is di­(2-propylheptyl) phthalate (DPHP), a high molecular weight (C10) phthalate plasticizer (Table S2) with manufacturing and/or import quantity in the European Union (EU) of ≥100 000 to <1 000 000 tons/year (Table S1). DEHP and DPHP are used foremost in polyurethane (PUR) and polyvinyl chloride (PVC) (soft) polymers in concentrations typically ranging between 2 and 35%,^17^ which is higher than that of other plastic additives.^4^ Both DEHP and DPHP (Table S2) have been prioritized as high-release potential PVC plasticizers of high and medium hazard concern, respectively.^18^ The emerging contaminant DPHP is foremost intended for exterior applications,^19^ where, over the course of the product’s life cycle, soils and sediments become important sinks for hydrophobic phthalates, migrating from the plastic matrix^20,21^ and emitted via wastewater effluents and sludge.^22^ Despite significantly reduced manufacture and use, DEHP emissions will remain high for decades, considering the lifetime of plastic waste.^23^ DEHP has been shown to be present in soils across all studies and land use categories^24^ at up to 264 mg/kg,^25^ but for DPHP in soil, there are no data. Likewise, DEHP concentrations in sediments have been shown to reach 19 mg/kg^26^ and pose risks to benthic biota.^26,27^ No data exist for DPHP concentrations in sediment/soil, but the correlation between occurrence and behavior between restricted and substitute plasticizers^27^ predicts comparable environmental levels for DEHP and DPHP in the future. In waterbodies, DEHP and DPHP have both shown 100% detection frequency in riverine suspended matter at up to 2 mg/kg and 1.4 mg/kg, respectively, whereas a 10-fold increase in DPHP concentrations was recorded in 2017 compared to 2005.^28^ In the EU, DEHP has been officially recognized as “Toxic to Reproduction” and “Endocrine Disrupting”. It is a reproductive toxicant 1B – H360FD. For DPHP, no harmonized classification is currently available (Table S1), and its assessment of “Endocrine Disrupting” is ongoing. Increasing environmental concentrations require targeted risk reduction measures also for the natural environment since most phthalate hazard data concern human health,^15^ not respecting the aims of the One Health approach.^29^ At the same time, exposure of wildlife to plastic additives is significant: restricted DEHP continues to be the predominant measured phthalate in all the studied individuals of marine mammals,^30^ and the first data on DPHP in wildlife appear.^31^ Literature search (10.02.26) in Clarivate WoS (by keywords di­(2-propylheptyl) phthalate AND toxic* in “All Fields”) gave 23 hits, of which only 2 regarded ecotoxicological impact, namely, on *Daphnia magna* ^32^ and amphibian *Xenopus laevis*.^33^ No data are available on potential terrestrial hazards of DPHP. For comparison, an analogous search for DEHP (di­(2-ethylhexyl) phthalate AND toxic*) gave 3213 hits. The effects of DEHP on terrestrial biota have mainly been studied in plants and earthworms, but hazard data for other soil invertebrates are limited.^26,34^ Understanding of the impact of phthalates and more so of emerging substitute phthalates, such as DPHP, on environmental organisms is, however, limited, which is concerning considering its industrial importance as a plastic additive and significantly increasing environmental concentrations.^15,28^ To improve the current hazard knowledge on plastic additives, the goal of this study was to evaluate the long-term hazard of high-volume substitute plasticizer DPHP and compare it to that of restricted DEHP. As plastic pollution affects all ecosystems, representatives of both aquatic (*D. magna*) and terrestrial arthropods (*Tenebrio molitor* and *Porcellio scaber*) were used for hazard evaluation. We hypothesized that the substitute plasticizer DPHP would induce lower hazard to the aquatic and terrestrial test organisms than the restricted DEHP.

## 2 Materials and Methods

### 2.1 Chemicals and Labware

The studied plasticizers were di­(2-propylheptyl) phthalate (DPHP) (BLDpharm, CAS No. 53306-54-0, 99,5%) and restricted di­(2-ethylhexyl) phthalate (DEHP) (Sigma-Aldrich, CAS No. 117-81-7, 99,5%). Ethyl acetate (VWR Chemicals BDH, HiPer Solv CHROMANORMUltra Plus; CAS 141–78–6, min 99,9%) and acetone (Sigma-Aldrich, CAS No. 67-64-1) were used as the solubilizers for plasticizers. Plasticizer isotopes diethyl phthalate (DEP) (CAS No. 93952-12-6) and dibutyl phthalate (DPP) (CAS No. 358730-89-9) were used in the GC–MS analyses of phthalate quantification. Plastic labware was avoided where possible. Glassware was prewashed with alkaline detergent Extran MA 01.

### 2.2 Impact of DEHP and DPHP on Aquatic Biota

#### 2.2.1 Spiking the Sediment for Aquatic Toxicity Exposures

*D. magna* was exposed to the plasticizers via a spiked sediment. Beach sand was used as the sediment. Prior to use, the sand was washed with 0.1 M HCl, rinsed with tap water, and dried. Spiking was performed at 25 mg and 50 mg plasticizer/kg sediment at 20% ethyl acetate. The solvent was evaporated at 60 °C for 2 h. Solvent control sediment was prepared analogously. Potential residual solvent in the sediment was quantified by headspace gas chromatography with flame ionization detection (HS-GC-FID) (*n* = 3).

#### 2.2.2 Experiments with Aquatic Crustacean *D. magna*

Chronic exposure of *D. magna* was conducted according to OECD211 ^35^ guidelines with small modifications as described hereafter. To evaluate the impact of plasticizers, parental survival, size, and reproduction (time to first brood, total number of broods, and number of offspring/survived female) were applied as toxicity end points. The impact was assessed over two generations (F0 and F1). Natural water was used as the exposure medium. The water was collected from Lake Ülemiste (Tallinn, Estonia), the main part of Tallinn’s water supply. Immediately after collecting, the water was filtered with 0.45 μm cellulose nitrate filters (Sartorius) and stored at +4 °C. Before toxicity exposures, the water was aerated for 1 h. Physicochemical parameters of natural water were the following: pH = 7.98, conductivity (20 °C) 437 μS/cm, hardness 2.30 mmol/L, 20 mg Cl^–^/L, 36 mg SO_4_^2–^/L, 2.79 mg K/L, 78.1 mg Ca/L, 8.43 mg Mg/L, 11.5 mg Na/L, 2.4 mg N/L, 0.062 mg P/L. For the exposure, one *D. magna* neonate (<24 h) on 10 g of sediment and 50 mL of natural water was used per parallel. Daily, *Pseudokirchneriella subcapitata* at 1.0 × 10^5^ cells/mL was added in each parallel as food. Exposure of each generation lasted for 21 days and was conducted at 21 ± 1 °C at 1200 lux at 16 h/8 h light/dark. The offspring were counted and removed daily. The exposure medium was not changed but was aerated daily. For the F1 generation exposures, at least 10 randomly chosen neonates were transferred to the respective samples. F1 exposures were started at the end of F0 exposures (at day 20–21). The exposure was conducted as described for F0 generation. For either generation, 2–5 independent experiments (à 7–13 technical surviving parallels) have been considered. Detailed replicate information is provided in Table S3.

### 2.3 Impact of DEHP and DPHP on Terrestrial Biota

#### 2.3.1 Spiking of Food and Soil for Terrestrial Toxicity Exposures

Stock solutions of 2 g of DEHP/L and 2 g of DPHP/L for woodlice and 20 g of DEHP/L for mealworms were prepared in acetone. These were used to prepare the working solutions, which were added to a) the dry soil to achieve the final concentrations of 5, 50, and 100 mg DEHP/kg dry soil and 5, 50, 200, and 1000 mg DPHP/kg dry soil for woodlice experiments or b) dry bran to achieve the final concentrations of 5, 50, 500, and 1000 mg DEHP/kg dry bran for mealworm experiments. When preparing the soil, 6.4 mL of acetone solution with the appropriate concentrations of DEHP or DPHP was added to 100 g of Lufa 2.2 soil for each treatment to ensure that the acetone content was the same in all treatments (5%, v/w). The soil with acetone was placed in the ventilation hood and left overnight to allow the acetone to evaporate. The next day, water was gradually added to the soil to achieve a water-holding capacity (WHC) of 30%. In the control group, organisms were only exposed to Lufa 2.2 soil with 5% acetone (which was also vaporized in the bonnet). In the case of the bran preparation, 5 g of bran with the previously added acetone solution with DEHP was mixed with an additional 95 g of untreated dry bran to achieve the selected final test concentrations. A positive control was also used in the experiment with the mealworms. For this purpose, stock solution of 10 g CdCl_2_/L was prepared in H_2_O. To achieve a final concentration of 5000 mg Cd/kg dry bran, we added 50 mL of stock solution to 100 g of dry bran. The bran with the added CdCl_2_ solution in water was placed in the laboratory oven (Kambi, Slovenia) and left for 2–4 h to remove all the water.

#### 2.3.2 Experiments with Woodlice *P. scaber*

Woodlice were exposed according to our standard protocol.^36^ Before the experiments, 30 mL of the liquid plaster mixture was poured into the 100 mL glass jars to dry. On the day of the experiment, water was poured over the plaster for 1 h to soak up the water, and the remains were poured out. The plaster is used to maintain the moisture of the soil. 20 g of the moist test soil was placed into jars, and five woodlice were placed in each jar. Five glass jars were prepared for each treatment (control and DEPH/DPHP treatments). A piece of dry, sterilized common hazel (*Corylus avellana*) leaf (∼100 mg) was added to each jar as an additional food. The moisture content of the soil was checked every 3 days by weighing the entire jar and adjusting it to an initial moisture content of 30% WHC. The hazelnut leaves were replaced every 5 days. Exposure lasted 2 weeks at 20 ± 2 °C in the dark. After the experiment, the survival of woodlice was analyzed. The immune response was assessed by analyzing selected immune parameters (hemocyte viability and proportion of the three major hemocyte types) in the hemolymph of individual organism according to Dolar et al.^37^ In the rest of the body, biochemical parameters related to neurotoxicity (AChE, acetylcholinesterase),^38^ detoxification (GST, glutathione S-transferase)^39^ and metabolism (ETS, electron transfer system)^40^ were measured. The methods are described in detail in the Supporting Information. Detailed replicate information is provided in Table S4.

#### 2.3.3 Experiments with Mealworms *T. molitor*

Mealworms were exposed as described in Jemec Kokalj et al.^41^ with minor modifications. The larvae of the mealworm *T. molitor* (60–90 mg) were obtained from our in-house laboratory culture. Approximately 30 g of the test wheat bran with phthalates was placed in a 500 mL plastic container. Moist paper towels (80 mm × 110 mm) were placed on top of the bran. All concentrations and controls were performed in 9 replicates (containers) for DEHP and 6 replicates for DPHP. Each container contained 5 larvae. This means that each of the end points presented (growth and moult) is based on 9 data points for DEHP and 6 data points for DPHP. The test vessels were incubated at 20 ± 1 °C in a 16:8 light/dark cycle. As toxicity end points, organism mass (larvae and pupae separately), mortality (each adult stage separately), moult, and occurrence of different developmental stages were measured. The method for following moult, animal growth, and pupae and larvae emergence is described in detail in the Supporting Information. Detailed replicate information is provided in Table S4.

### 2.4 Plasticizer Quantification in the Exposure Media

Nominal plasticizer concentrations in the spiked sand were verified by GC–MS at different time points (*t* = 0, 6, 21 days). For GC–MS analysis, 5 mL of ethyl acetate and a mix of three internal isotope standards (DEHP, DEP, and DPP), each at 100 μg/L, was added to the lyophilized 0.5 g of spiked and 2 g of blank sediment, and plasticizer extraction was enhanced by 15 min in UH-bath (Elmasonic P 30H, 480/400 W). The obtained extracts were purified in Si-gel (Merk Kieselgel 60 reinst 70–230 mesh) (washing with 3 mL of ethyl acetate and 2 mL of the extract), from where 1 mL of purified extract was collected for GC–MS analysis. Results were calculated with MassHunter software MS Quantitative Analysis (2019) program via the isotope internal standard calibration using the SIM method, as in the modified CEN/TS 16183:2012.^42^ Soil samples were collected from the batch of test soil that was distributed in test jars for woodlice testing. They were stored at −20 °C until analyzed. Analysis was not done on the soil after the experiments (2 weeks), because the soil contains animal feces and leaf remnants, which changes the soil mass, and hence, the analysis is not relevant.

### 2.5 Quality Control and Quality Assurance

The nominal phthalate concentrations in the sediment (sand) and soil were confirmed by GC–MS (*t* = 0), and since both DPHP and DEHP are classified as potentially biodegradable, their concentrations in the spiked sand were measured over 21 days. Across the time-points (0, 6, and 21 days), the measured plasticizer concentrations differed from the nominal by up to 20% (DEHP) and up to 13% (DPHP). The median differences, though, were 4% (DEHP) and 2% (DPHP) (Table 1). No plasticizers were detected in the blank control samples (nonspiked soil/sand). The limit of quantification for the GC–MS method was 0.14 mg/kg dwt for DEHP and 0.31 mg/kg dwt for DPHP. Residual solvent quantification by HS-GC-FID showed ethyl acetate concentrations in all the analyzed sediments (*n* = 3) of *D. magna* below the limit of quantification (0.01 g/kg) and in accordance with recommended solvent concentrations of below 20 μL/L.^43^ To avoid/minimize the adsorption of phthalates, plastic labware was avoided. In *D. magna* assays, pipet tips were the only plastic consumables used, and all the glassware was prewashed with alkaline detergent Extran MA 01. Mealworms *T. molitor* were exposed to plastic containers due to technical reasons.

**Table 1:** Nominal and Measured^a^ (GC–MS) di­(2-ethylhexyl) Phthalate (DEHP) and di­(2-propylheptyl) Phthalate (DPHP) Concentrations (mg/kg dwt) in the Spiked Soil as Prepared for *P. scaber* Exposure and in the Sediment (Sand) as Prepared for *D. magna* Exposures During up to 21 days (*t* = 0, 6, and 21)

		DEHP			DPHP			
		mg/kg soil						
nominal	*t* = 0	5	50	100	5	50	200	1000
measured	*t* = 0	4.0 ± 0.1	44 ± 4.8	97 ± 1.3	4.9 ± 0.2	57 ± 3.9	194 ± 1.5	982 ± 70

		mg/kg sediment (sand)			
nominal	*t* = 0	25	50	25	50
measured	*t* = 0	22 ± 5.3	54 ± 6.6	25 ± 6.6	53 ± 9.0
	*t* = 6	25 ± 3.3	48 ± 2.7	24 ± 2.5	50 ± 1.6
	*t* = 21	20 ± 4.9	49 ± 5.4	24 ± 5.8	48 ± 8.2

a The measured concentrations are expressed as AVG ±SD (*n* = 2 for soil and *n* = 3–8 for sediment (sand)).

### 2.6 Data Analysis

For data analyses and visualization, DataTab (Graz, Austria)^44^ and OriginPro v2023b software (OriginLab, Northampton, MA, USA) were used. Normal distribution (*p* > 0.05) of the data was analyzed using Kolmogorov–Smirnov (Lilliefors Corr.), Shapiro–Wilk, and Anderson–Darling tests. Equality of variance of the data was analyzed with Levene’s test. For hypothesis testing, depending on the data normality and homoscedasticity, either parametric one-way ANOVA (Tukey’s posthoc) or nonparametric Kruskal–Wallis (Dunn-Bonferroni posthoc) or Mann–Whitney U was used. Pairwise comparison was performed with the solvent control (ethyl acetate for *D. magna* and acetone for *P. scaber* and *T. molitor*).

## 3 Results

### 3.1 Plasticizer Concentration in the Exposure Media

For toxicity evaluations, nominal concentrations of 5–1000 mg of phthalate/kg of medium were used to cover a range of potential exposure settings. No significant concentration decrease in time was recorded: after 21 days of abiotic *D. magna* exposure, concentrations, on average, decreased less than 10% for both plasticizers (Table 1). Thus, nominal concentrations can be referred to throughout the paper.

### 3.2 Impact of DEHP and DPHP on Aquatic Biota

#### 3.2.1 Water Flea *D. magna*

The water flea *D. magna* was exposed to DEHP and DPHP in the F0 and F1 generations for 21 days in each. In line with the OECD211 ^35^ guidelines, only the tests where performance criteria for the negative (untreated) control survival and fertility were met were included in the analysis. Ethyl acetate was used for solubilizing the phthalates, and the impact of phthalates is presented upon pairwise comparison with the solvent control. However, intergenerational differences (*p* ≤ 0.05) within the treatment groups (Tables 2 and S3) have also been considered. According to OECD211 performance criteria, the mortality of the parental daphnids in the control should not exceed 20% for the test to be valid. In the F0 generation, survival in all the treatment groups was 98%, with the exception of the 50 mg/kg phthalate groups, where it was 94% and 84% for DEHP and DPHP, respectively. In F1 generation, survival rates of 100% (control, 50 mg DEHP/kg), 97% (25 mg DEHP/kg), 90% (solvent control, 50 mg DPHP/kg), and 87% (25 mg DPHP/kg) were recorded. The size of F0 parental organisms at day 21 was smaller (*p* < 0.05) in both 50 mg/kg phthalate groups: daphnid body length (AVG ±SD) was 3.55 ± 0.20 mm in the solvent vs 3.42 ± 0.19 mm and 3.45 ± 0.20 mm in DEHP and DPHP, respectively (Figure 1, Table S5). Intergenerational analysis showed that parental size in all of the treatment groups (except for the 50 mg of DEHP/kg group) had decreased in F1 generation (Table 2). Parental daphnids in the 25 mg DEHP/kg group underwent the most significant decrease in body length (Figure 1, Table 2). In the F0 generation, reproduction was delayed in 50 mg/kg phthalate groups: in the solvent, reproduction started at day 9.3, but in phthalate groups at day 10.1 (DEHP) and 10.2 (DPHP) (Figure 1). In the F1 generation, delayed reproduction persisted only in the 50 mg/kg DPHP group: 8.9 vs 10.7 days to first brood was recorded for solvent and DPHP, respectively (Figure 1). In the F1 generation, days to first brood were also delayed in 25 mg/kg phthalate groups, but this was due to intergenerational reproduction delay (*p* < 0.05) within the solvent group (Table 2). Delayed reproduction at 50 mg DPHP/kg was the most significant persistent effect for this toxicity end point and was also reflected in the reduced (*p* < 0.05) number of broods in F1:3.67 vs 4.18 broods/female for 50 mg DPHP/kg and solvent group, respectively (Figure 1). The number of live offspring/survived females was higher in the control (61.7 ± 8.97) compared to the solvent control (55.5 ± 15.9) (*p* < 0.05) in the F0 generation. In the F1 generation, the number of offspring/female in the two controls was no longer different (Figure 1 and Table 2) but 48.2 ± 15.6 offspring/female in the 25 mg DEHP/kg group was significantly lower than that of the solvent (62.8 ± 20.6).

**Table 2:** Intergenerational Changes (Comparison of F1 With F0) in Size (Body Length) and Reproductive Parameters (days to First Brood, Number of Broods, and Offspring/Female) of Parental *Daphnia magna*

	*Daphnia magna* size	days to 1st brood	number of broods	offspring/female
	change (%)	change (%)	change (%)	change (%)
control	4,9^c^	–0,1	4,6	–0,3
solvent	4,8^c^	4,1^b^	–10^a^	–13
DEHP	8,7^c^	–0,1	2,6	9,5
25 mg/kg				
50 mg/kg	0,3	12^b^	–1,1	–10
DPHP	4,3^c^	2,3	–1,0	8,4
25 mg/kg				
50 mg/kg	3,8^c^	–5,3	6,7	–1,5

a *p* < 0.05.

b *p* < 0.01.

c *p* < 0.001 (Mann–Whitney U) indicates differences from the respective values of the F0 generation. Solvent–ethyl acetate; DEHP-di­(2-ethylhexyl) phthalate; DPHP- di­(2-propylheptyl) phthalate. Detailed values (AVG ±SD) are available in Table S5.

**Figure 1:**
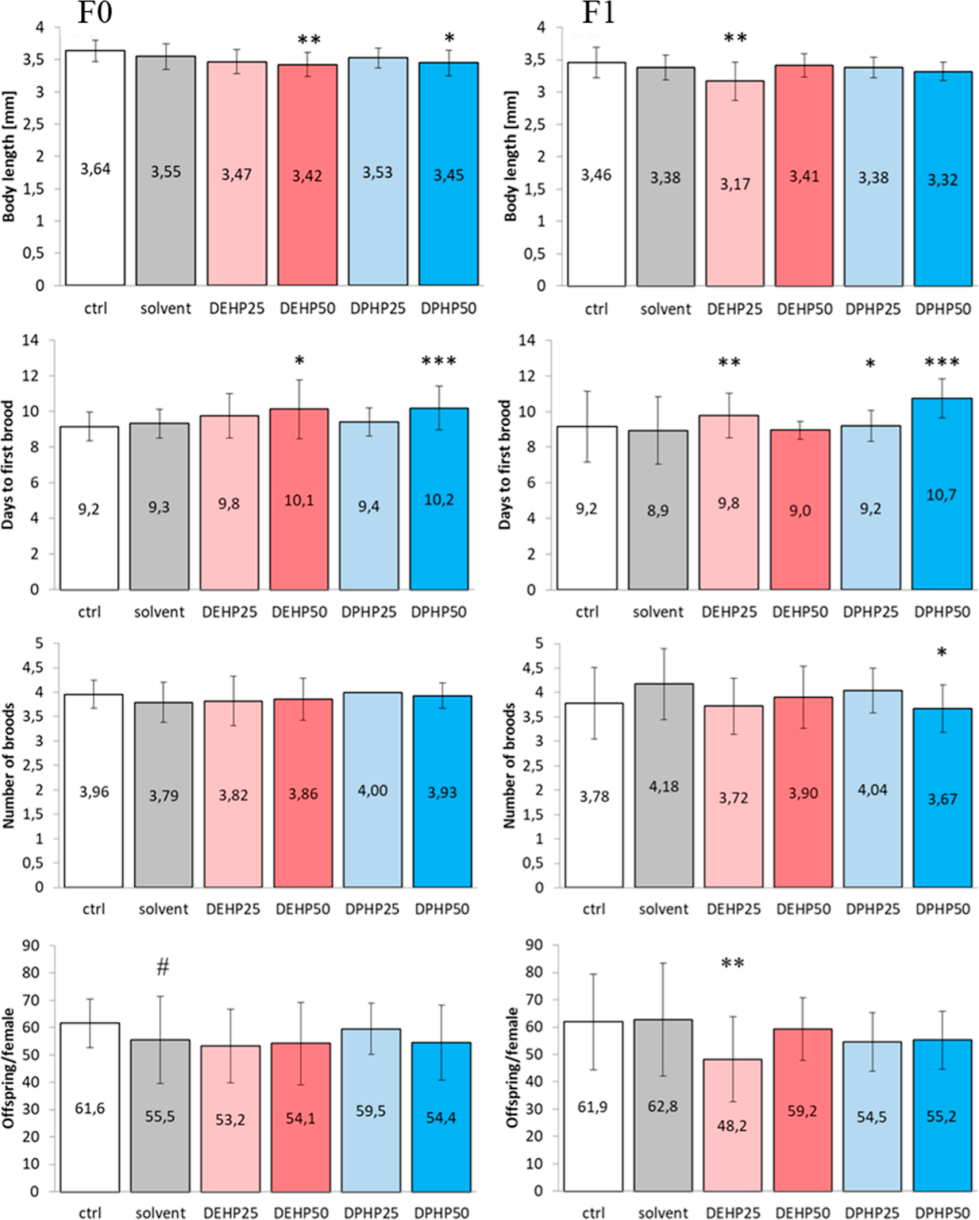
Impact of di­(2-ethylhexyl) phthalate (DEHP) and di­(2-propylheptyl) phthalate (DPHP) on the size (body length) and reproduction (days to first brood, number of broods, and offspring/female) of *Daphnia magna* in F0 (left column) and F1 (right column) generation. Exposure concentrations were 25 or 50 mg phthalate/kg sediment. The asterisks indicate difference (*p < 0.05; **p < 0.01; ***p < 0.001 (Mann–Whitney U)) from the solvent (ethyl acetate) control. # indicates the solvent ethyl acetate control is significantly (*p* < 0.05) different from the nonspiked control (ctrl). The data are presented as AVG ±SD (*n* = 2–5). Control-normalized plots for *D. magna* are shown in Figure S2.

### 3.3 Impact of DEHP and DPHP on Terrestrial Biota

#### 3.3.1 Mealworms *T. molitor*

Neither DEHP nor DPHP affected the survival of mealworms during 8 weeks of exposure. The mortality of control groups was 13% for DEHP and 8% for DPHP. The mortality of all exposure groups was below 20%, which is a common threshold allowed for controls in chronic tests with terrestrial invertebrates. The mortality of mealworms in Cd exposure (5000 mg of Cd/kg of food) was 27%.

#### 3.3.2 Growth and Moulting of Larvae

The growth of larvae was generally not affected by DEHP exposure except for a significant induction of growth observed at 500 mg/kg after 4 weeks (*p* < 0.05). In this case, the growth was increased by 13% in comparison to the control (*p* < 0.05). The moulting of larvae was also not changed upon DEHP exposure. Similarly, no effects of DPHP on the growth and moulting of larvae were noted. Positive control, 5000 mg Cd/kg food, significantly affected the growth of larvae (*p* < 0.05) (decreased by 33%), but did not change moulting (Figure 2).

**Figure 2:**
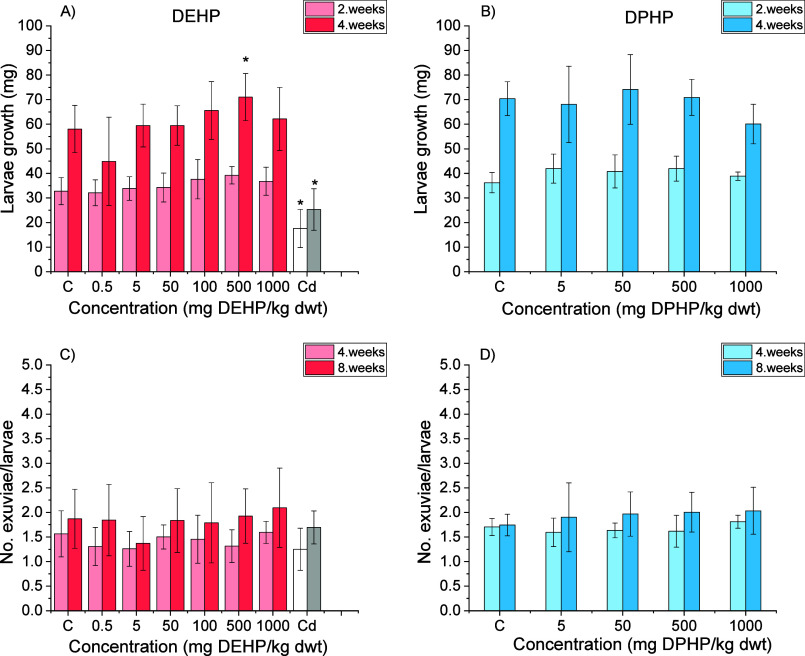
Growth (A,B) and moulting (C,D) of *Tenebrio molitor* larvae exposed to di­(2-ethylhexyl) phthalate (DEHP) and di­(2-propylheptyl) phthalate (DPHP) for 8 weeks. The concentration of the positive control chemical Cd was 5000 mg Cd/kg food (bran). White and gray bars represent Cd treatment (2 and 4 weeks, respectively, for growth and 4 and 8 weeks, respectively, for moult). The asterisks indicate a difference (*p < 0.05; Kruskal–Wallis ANOVA, Dunn’s test) from the solvent (acetone) control. The data are presented as AVG ±SD (*n* = 9).

#### 3.3.3 Development of Mealworms

The development of pupae and adults was followed during the 8 week exposure. No differences between the negative control (nonspiked food) and DEHP and DPHP exposure concentrations were observed (Figure S1). The final emergence of pupae and adults after 8 weeks was not different between the control and DEHP and DPHP exposures (Figure 3). The positive control chemical cadmium significantly impaired the development of mealworms (*p* < 0.001), i.e., reduced the emergence of pupae and adults by 60% and 67%, respectively, compared to the negative control group (Figure 3).

**Figure 3:**
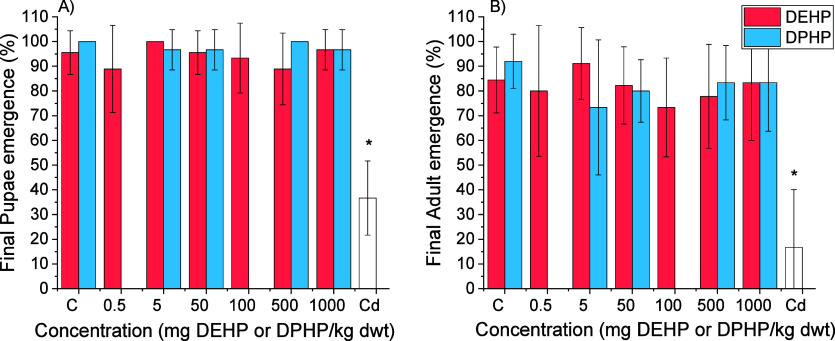
Final emergence of *Tenebrio molitor* pupae (A) and adults (B) after 8 weeks of development upon exposure to di­(2-ethylhexyl) phthalate (DEHP) and di­(2-propylheptyl) phthalate (DPHP). The concentration of the positive control chemical Cd was 5000 mg Cd/kg food (bran). The asterisks indicate a difference (*p < 0.05; Kruskal–Wallis ANOVA, Dunn’s test) from the solvent (acetone) control. The data are presented as AVG ±SD (*n* = 9). Control-normalized plots for *T. molitor* are shown in Figures S3 and S4.

### 3.4 Woodlice *P. scaber*

No statistically significant changes in the survival of woodlice exposed to up to 100 mg/kg of DEHP and 1000 mg/kg of DPHP were observed compared to controls.

#### 3.4.1 Immune Response

We observed no change in hemocyte viability nor the proportions of different hemocyte types in woodlice exposed to DEHP up to 100 mg/kg and DPHP up to 1000 mg/kg (Figure 4).

**Figure 4:**
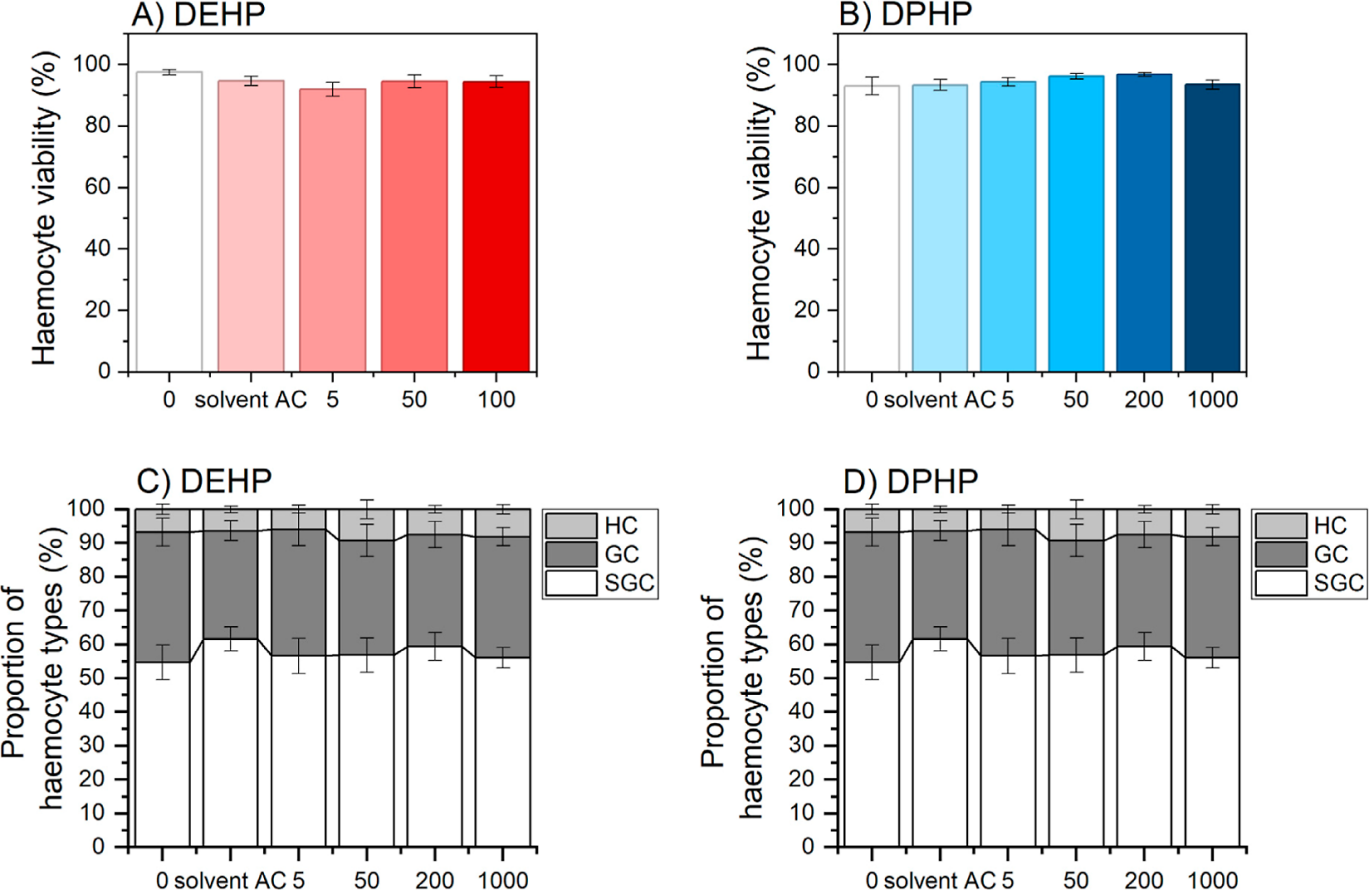
Immune response of woodlice *Porcellio scaber* exposed to di­(2-ethylhexyl) phthalate (DEHP) (A,C) and di­(2-propylheptyl) phthalate (DPHP) (B,D). HC-hyalinocytes, SGC-semigranulocytes, GC-granulocytes. Viability of hemocytes is shown. The data are presented as AVG ±SD (*n* = 10). Control-normalized plots for *P. scaber* are shown in Figure S5.

#### 3.4.2 Enzyme Activities

Significant differences in enzyme activities were observed in DEHP exposure (Figure 5A,C,E). AChE activity was increased at 50 and 100 mg/kg (*p* < 0.01) exposure concentrations (Figure 4A) by ∼ 70%, while GST activity of woodlice was increased by 18% (Figure 5C), and ETS activity was decreased by 22% at the highest concentration tested (100 mg DEHP/kg) (Figure 5E). In the case of DPHP, GST activity was increased at all the concentrations above 5 mg DPHP/kg (*p* < 0.05 for 5, 50, and 200 mg/kg, and *p* < 0.001 for 1000 mg/kg) (Figure 5D). The increase was by 90%, 78%, 63%, and 112% at 5, 50, 200, and 1000 mg/kg, respectively. However, no changes in AChE and ETS activities were observed compared with the control (Figure 5B,F).

**Figure 5:**
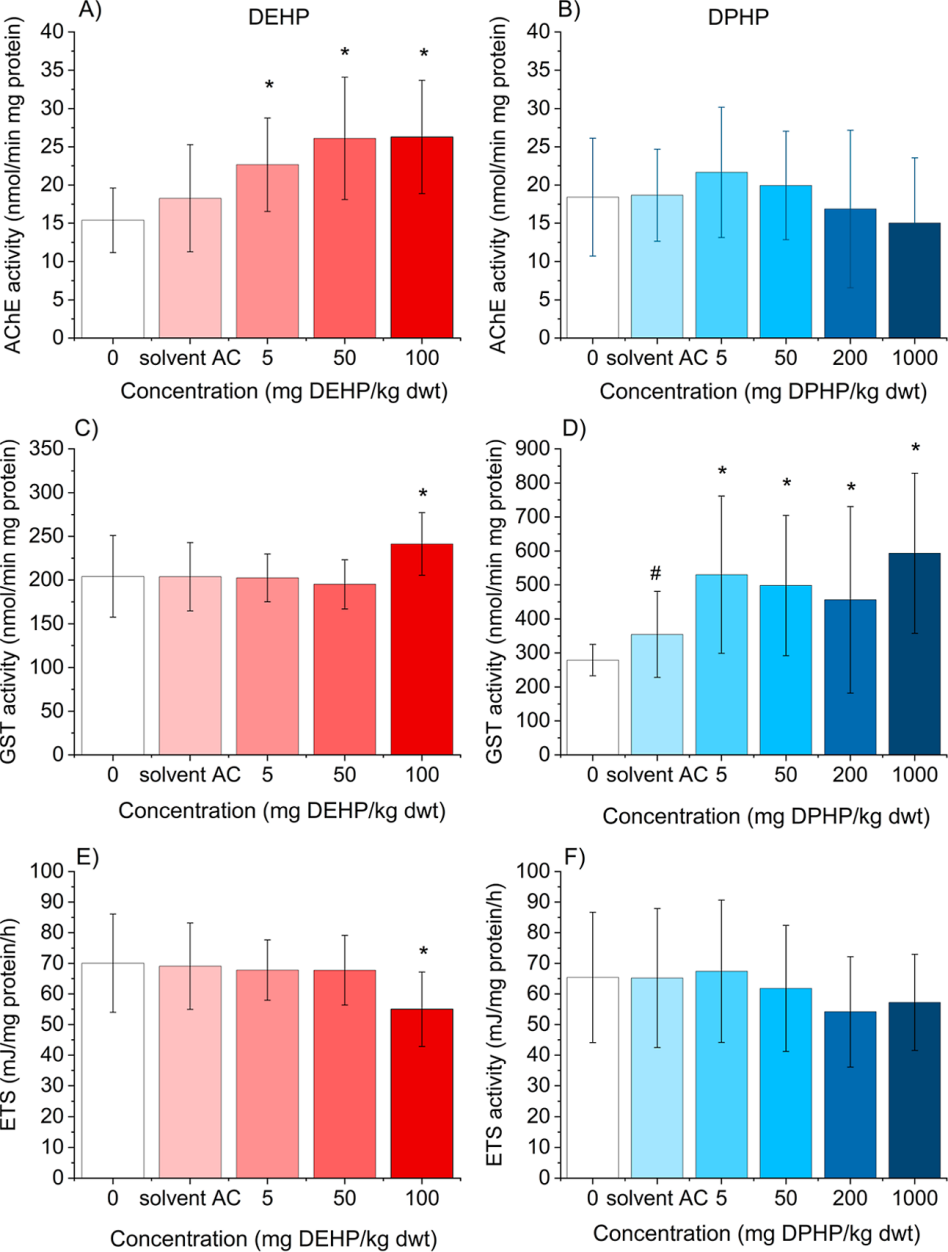
Activity of acetylcholinesterase (AChE) (A,B), glutathione S-transferase (C,D), and electron transfer system activity (ETS) (E,F) in the whole body of woodlice *Porcellio scaber* exposed to di­(2-ethylhexyl) phthalate DEHP (A,C,E) or di­(2-propylheptyl) phthalate DPHP (B,D,F) in spiked soil for 14 days. The asterisks (*) indicate a difference (*p < 0.05; Kruskal–Wallis ANOVA, Dunn’s test) from the solvent AC (acetone) control; # denotes statistically significant difference from the control (0) (Kruskal–Wallis ANOVA, Dunn’s test). The data are presented as AVG ±SD (*n* = 15 for DEHP, *n* = 10 for DPHP).

## 4 Discussion

In the current study, the ecotoxicological impact of two common plastic additives, restricted plasticizer restricted di­(2-ethylhexyl) phthalate (DEHP) and emerging substitute di­(2-propylheptyl) phthalate (DPHP), was studied with one aquatic and two terrestrial arthropod species. As sediments and soils are sinks for phthalates,^27,45^ for higher environmental relevance, organismal exposures were carried out via phthalate-spiked sediment/soil or food (bran). The degradation half-life for DEHP ranges from hours (photooxidation) to years (photo- and hydrolysis), whereas in sediment/soil, the biodegradation half-life is typically weeks to months.^46^ Biodegradation is the leading degradation process for phthalates in surface water, sediment, and soil, and low molecular weight phthalates are more easily degraded.^46^ In the current study, however, even during the longest continuous exposure of 21 days, differences between the nominal and measured concentrations in sediment (sand) were not higher than 20% (Table 1), in line with Brown et al.^32^

### 4.1 Impact of DEHP and DPHP on Aquatic Arthropods

Aquatic toxicity evaluation of hydrophobic organic compounds, such as phthalates, is challenging. Passive dosing methods^47,48^ could be suggested as the method of choice to avoid using solvents/dispersants that potentially induce adverse effects of their own.^49^ In the current study, *D. magna* exposure was conducted via spiked sediments using ethyl acetate for dispersing. Ethyl acetate is considered practically nontoxic to aquatic organisms,^50^ including in long-term settings^51^ for *D. magna* (21 day NOEC = 2.4 mg/L).^52^ However, in the solvent control, *D. magna* results showed lower fertility compared to the negative control in the F0 generation (Figure 1). Differently from the negative control, the offspring from F0 solvent control started to reproduce earlier in F1 (*p* < 0.01), leading to more broods than in F0 (*p* < 0.05) (Tables 2 and S5). To account for the potential influence of the residual ethyl acetate in the sediment,^53^ aquatic toxicity of DEHP and DPHP was analyzed in pairwise comparison with the solvent control.^54^

Both phthalates influenced parental *D. magna* body length as well as reproductive end points. In the F0 generation, at 50 mg/kg, both DEHP and DPHP induced delayed reproduction as well as 4% (DEHP) and 3% (DPHP) smaller body lengths of parental organisms. The onset of reproduction was delayed by 8% (DEHP) and 9% (DPHP). For DPHP, delayed reproduction persisted in the F1 generation and had increased to 20% (Table S5). Based on the scarce ecotoxicological data, on the example of *X. laevis* embryos, DPHP has been ranked less toxic and teratogenic than conventional low molecular weight phthalates (DBP and BBP) but more hazardous than substitute cyclohexanoate plasticizers (e.g., DINCH).^33^ Long-term endoplasmic reticulum stress-related and mitochondrial apoptosis and interfered cellular damage repair were proposed to induce malformations (developmental asymmetry) in *X. laevis* above ≥100 mg DPHP/L and mortality above 500 mg DPHP/L.^33^ LOEC (developmental toxicity) was 10 mg DPHP/L.^33^ In 21 day *D. magna* assay, comparative assessment of DPHP and DEHP showed no impact on organismal length nor reproduction at 1 mg/L^32^ but 3-generation-long exposure to 0.32 mg DEHP/L, depending on the initial population size, was projected to potentially lead to *D. magna* population collapse.^55^ Another study, however, reported 1.5-fold increased *D. magna* reproduction upon 14 day exposure to 0.39 mg/L DEHP, whereas parental organism body length was reduced.^56^ In risk assessment of endocrine disrupting chemicals, including DEHP, nonmonotonic dose–response has often been described,^57^ including for *D. magna*.^32,58,59^ Here, in *D. magna* assays, the lower DEHP concentration (25 mg/kg) induced effects in the F1 generation, whereas the higher concentration (50 mg/kg) did not (Figure 1). This phenomenon occurred only in the F1 generation and only for DEHP. Similar nonmonotonic dose–response was observed for 500 mg DEHP/kg-exposed *T. molitor* larvae-induced growth at 4 weeks. The hypothesis that lower concentrations of phthalates affect biological systems differently than can be predicted from higher concentration testing requires further research. Although *D. magna* chronic reproduction assay potentially underestimates endocrine-disrupting potential of chemicals,^60^ reproductive end points may nevertheless reflect altered amino-acid and energy metabolism, markers of phthalate exposure.^56,59,61,62^ Altered metabolism upon DEHP-exposure has also been demonstrated in vivo.^63^

### 4.2 Impact of DEHP and DPHP on Terrestrial Arthropods

Tested phthalates did not systematically affect moulting, growth, development, the survival of *T. molitor,* and survival of *P. scaber* at tested concentrations (up to 100 mg DEHP/kg, 1000 mg DPHP/kg). This indicates that neither phthalate was very toxic to test terrestrial arthropods. Woodlice also did not respond to exposure to DEHP or DPHP with a significant immune response, as is usually the case with chemical exposure.^64^ However, DEHP led to significant changes in enzyme activities, such as AChE and GST, and metabolic activity, measured as the ETS activity. This indicates a potentially neurotoxic effect (>50 mg/kg) and an induction of detoxification processes (>100 mg/kg) with simultaneously reduced metabolic activity. DPHP, on the other hand, induced changes only in the detoxification enzymes, but already at 5 mg/kg. It is not possible to infer why DPHP induced detoxification at lower concentrations than DEHP, as they are chemically similar. For some groups of phthalates, similar mechanisms of action have been proposed for vertebrates, including apoptosis, oxidative stress, genotoxicity, inflammation, and neuronal and metabolic disorders.^65–67^ However, we could not find any study comparing the toxicity of DEHP and DPHP. Overall, our results show that both phthalates trigger a certain physiological response in woodlice. Our results are consistent with the literature, where for terrestrial biota, exposure to DEHP has been shown to induce low/no effect at the organism level, even at g/kg levels. For example, the impact on growth, burrowing rate, and survival of earthworms *Eisenia* sp. and *Metaphire guillelmii* was recorded at 8000 mg/kg and 2000 mg/kg, respectively.^68^ In other studies, no impact on survival, burrowing rate, esterase activities, and oxidative stress in *Eisenia andrei* was detected in acute exposure to 20000 mg/kg soil.^34^ Comparable sensitivity to DEHP has been shown for springtails, as no acute toxicity has been shown for adult *Lobella sokamensis* at up to 20000 mg/kg and chronic toxicity for juvenile *Folsomia fimetaria* at up to 5000 mg/kg.^69^ Juvenile *Folsomnia fimetaria* was not affected by long-term exposure up to 5000 mg/kg^69^ but survival of adult *F. candida* was affected above 1000 mg/kg.^34^ On the other hand, similar to our study, several authors reported that even very low concentrations of DEHP caused various metabolic changes. Oxidative stress was induced in *Eisenia fetida* after 7 day exposure to 1 mg/kg^21,70^ -day exposure to 2.5 mg/kg^14,68^ -day and 28 day exposure to 50 mg/kg.^71^

## 5 Conclusions

This study showed that both phthalate plasticizers, restricted di­(2-ethylhexyl) phthalate DEHP and substitute di­(2-propylheptyl) phthalate DPHP, remained stable in the sand for 21 days, with concentrations fluctuating within ±20% of nominal values. An important limitation of the study was the use of solvents, which induced some effects in the aquatic crustacean *D. magna*. However, this has been considered in the data analysis. Both DEHP and DPHP affected parental growth and reproductive end points of *D. magna* at 50 mg/kg of sediment. DPHP-induced reproduction onset delay persisted and even progressed in the F1 generation. For terrestrial arthropods, mealworms *T. molitor* and woodlice *P. scaber*, no systematic effects on organismal end points were observed up to the highest concentrations tested. However, biochemical changes were observed for *P. scaber*, with both phthalates triggering detoxification processes. Furthermore, DEHP exposure led to changes in the neurotoxic biomarker AChE and altered metabolic activity. Results indicate that neither DEHP nor DPHP was highly toxic to the test organisms but rather triggered physiological responses and, in the case of *D. magna*, also the population shifts. Overall, we provided evidence that substituted high volume plasticizer DPHP showed biological effects comparable to DEHP, indicating that the substitute plasticizer can also induce measurable hazards to exposed organisms. Future studies should prioritize evaluating the potential environmental hazards of substitute plastic additives, which are often introduced into the market without comprehensive testing.

## Supplementary Material



## Notes

Additional details on *T. molitor* and *P. scaber* assays, legislative regulation of selected phthalates, characterization of DEHP and DPHP, total number of independent experiments (*D. magna, T. molitor, and P. scaber*), comparison of toxicity end points of *D. magna* in F0 and F1 generations, emergence of *T. molitor*, and control-normalized plots (*D. magna*, *T. molitor*, and *P. scaber*) (PDF)
