# Supplementary material for: High-Volume Plasticizer di(2-Propylheptyl) Phthalate DPHP Induced Ecotoxic Effects in Aquatic and Terrestrial Arthropods
Source: ACS Environ Au. 2026 Apr 5;6(3):395–405. doi: 10.1021/acsenvironau.5c00191 (PMC13195461; doi:10.1021/acsenvironau.5c00191)
Supplement: Supplementary file 1 [file vg5c00191_si_001.pdf]

Supporting Information for

The high-volume plasticizer di(2-propylheptyl) phthalate DPHP induced ecotoxic effects in aquatic and terrestrial arthropods

Anita Jemec Kokalj[1], Andraž Dolar[1], Taja Korpar[1], Aljona Lukjanova[2], Margit Heinlaan[2*]

[1]Biotechnical Faculty, University of Ljubljana, Jamnikarjeva 101, 1000 Ljubljana, Slovenia
[2]Laboratory of Environmental Toxicology, National Institute of Chemical Physics and Biophysics, Akadeemia tee 23, 12618 Tallinn, Estonia

*Corresponding author margit.heinlaan@kbfi.ee

Methods

Mealworms *Tenebrio molitor*

Moult was assessed as follows: each week, larvae were marked with a black permanent marker pen (stripe on the dorsal side). The next week larvae were inspected and those that had no stripe were assumed to have moulted. The moult was calculated as the total number of moults per larvae after 4 and 8 weeks. Moult was corrected for the mortality of larvae.

Average animal mass per treatment was recorded as the joint mass of larvae divided by the number of live larvae at a certain time point. Growth was calculated as the difference in average animal mass after a certain period (2 and 4 weeks) and the beginning of the experiment.

Pupae and adult emergence were followed during the entire duration of the experiment. The cumulative number of newly emerged pupae and adults each week was divided by the total number of larvae exposed initially and expressed as the share of emerged pupae/adult until certain week. The total mortality was expressed jointly for all mealworm stages (larvae, pupae and adult) each week.

Woodlice *Porcellio scaber*

Immune parameters

The haemolymph of individual *P. scaber* was collected by puncturing the intersegmental membrane with a sterile syringe on the dorsal side between the 5th and 6th segment and gently squeezing the animal to obtain a small drop of haemolymph. Five microlitres of haemolymph were collected using a glass micropipette (Brand) and immediately diluted 1:5 with Dulbecco's phosphate buffered saline buffer (DPBS, pH=7.1–7.5) and 0.4% trypan blue, which stains dead

haemocytes while viable haemocytes remain un-stained. Ten microlitres of the haemocyte suspension was pipetted on to a Neubauer haemocytometer to evaluate the THC, haemocyte viability and the proportion of the three major haemocyte types (SGC: semigranulocytes, GC: granulocytes, HC: hyalinocytes) under Axio Imager Z1 light microscope (Zeiss, Germany). The proportion of the three haemocyte types was determined by counting the viable cells using the DIC microscopy technique.

Biochemical parameters

The body of the animals was homogenised in 800 μL of 100 mM potassium phosphate buffer (PPB) (pH=7.0) + 0.5% Triton X-100 using a T10 IKA Ultra-turrax homogeniser for 1 minute. The homogenates were centrifuged for 15 minutes at 20,817g at 4°C. All the used chemicals were of analytical grade from Sigma-Aldrich, Germany.

The activity of acetylcholinesterase (AChE) was measured in the supernatant according to the Ellman method (1) with minor modifications. The reaction mixture per well was: 50 μL of phosphate buffer saline (PPB) (50 mM, pH=7.0), 50 μL of supernatant, 100 μL of Ellman's reagent with acetylthiocholine chloride (ACh-Cl), containing 0.1148 mM of DTNB and 1 mM ACh-Cl as the final concentrations in the well. The working concentrations of 5,5′-Dithiobis(2-nitrobenzoic acid) and ACh-Cl were prepared immediately before use. The reactions were followed photometrically using Cytation 3 imaging reader (Biotek, USA) at 405 nm at 25°C for 15 minutes. All samples were measured in triplicates. AChE activity was expressed in nmol of hydrolysed ACh-Cl min^{-1} mg^{-1} protein (extinction coefficient, $ε405=13,600\ M^{-1}cm^{-1}$).

The activity of glutathione S-transferase (GST) was measured in the supernatant according to the method of Habig et al. (2) with minor modifications. The reaction mixture per well was: 50 μL of the supernatant, 50 μL of 0,1-chloro-2,4-dinitrobenzene (CDNB) (4 mM) and 100 μL of reduced glutathione (GSH) (2 mM). For blank reactions, the supernatant was replaced with 50 μl PPB (100 mM, pH=7.0). The reaction was followed spectrophotometrically at 340 nm at 25°C for 20 minutes. GST activity was expressed in nanomoles of conjugated reduced GSH min^{-1} mg $protein^{-1}$ (extinction coefficient, $ε340 = 9600\ M^{-1}\ cm^{-1}$).

The activity of electron transfer system (ETS) was measured according to De Coen and Janssen (3) at 490 nm and 25°C for 5 minutes. A volume of 300 μL of supernatant was added to 150 μL of homogenisation buffer (HB) (0.1 M KH_2PO_4, 0.1 M NaH_2PO_4, 830 μM $MgSO_4$, 0.15% (w/v) polyvinylpyrrolidone (PVP) and 0.2% (w/v) Triton X-100), mixed and centrifuged at 3,000 g for 10 minutes at 4°C. The reaction was measured in 96-well microplates, by mixing 50 μL of sample and 150 μL of buffer substrate solution (BSS) (0.1 M KH_2PO_4, 0.1 M NaH_2PO_4, 1.8 mM nicotinamide adenine dinucleotide, 0.28 mM nicotinamide adenine dinucleotide phosphate and 0.27 % (w/v) Triton X-100 and initiated by 100 μL of 3.95 mM Iodonitrotetrazolium chloride

(INT). ETS activities were calculated using a molar extinction coefficient of formed formazan ($\varepsilon490$=15,900 M^{-1} cm^{-1}), expressed in mJ mg $protein^{-1}$ h^{-1}.

Protein concentrations in the supernatant were determined using the BCA™ protein assay kit (Pierce, Rockford, IL, USA). After 30 minutes of incubation at 37°C, the proteins were measured at 562 nm. The concentration of proteins in the sample was calculated from the standard curve for bovine serum albumin (BSA) (25-2000 μM).

Table S1. Legislative regulation of restricted/banned ortho-phthalate plasticizers and selected substitute plasticizers in the EU

Plasticizer	CAS No	Manufacturing and/or import quantity in EU [tonnes per annum]	CLP Regulation No 1272/2008	REACH, Annex XIV	REACH, Annex XVII	Directive 2004/37/EC (CMRD)	Regulation 10/2011/EU	Regulation (EU) 2022/1616	Directive 2007/42/EC	Directive 92/85/EEC	Directive 94/33/EC	Directive 2011/65/EU	Regulation 1223/2009/EC	Regulation 1107/2009/EC	Directive 2010/75/EU	Directive 2008/105/EC
**DIBP**	84-69-5	≥ 1	Repr. 1B - H360Df	Auth. - entry 7	Restr. - entry 51	Restricted	-	-	-	Restricted	Restricted	Restricted	Banned	Banned	-	-
**DEHP**	117-81-7	≥ 10 000 to < 100 000	Repr. 1B - H360FD	Auth. - entry 4	Restr. - entry 51	Restricted	Restricted	Restricted	-	Restricted	Restricted	Restricted	Banned	-	Restricted	Restricted
**DBP**	84-74-2	≥ 1 000	Repr. 1B - H360Df<br>Aq. Acute 1 - H400	Auth. - entry 6	Restr. - entry 51	Restricted	Restricted	Restricted	-	Restricted	Restricted	Restricted	Banned	Banned	-	-
**BBP**	85-68-7	≥ 1 to < 10	Repr. 1B - H360Df<br>Aq. Acute 1 - H400<br>Aq. Chronic 1 - H410	Auth. - entry 5	Restr. - entry 51	Restricted	Restricted	Restricted	-	Restricted	Restricted	Restricted	Banned	-	-	-
**DCHP**	84-61-7	≥ 100 to < 1 000	Repr. 1B - H360D<br>Skin Sens. 1 - H317	-	-	Restricted	-	-	Restricted	Restricted	Restricted	-	Banned	-	-	-
**Alternative plasticizer**																
**DPHP**	53306-54-0	≥ 100 000 to < 1 000 000	-	-	-	-	Restricted	Restricted	-	-	-	-	-	-	-	-
**DEHT**	6422-86-2	≥ 100 000 to < 1 000 000	-	-	-	-	Restricted	Restricted	-	-	-	-	-	-	-	-
**DINCH**	166412-78-8	≥ 10 000	-	-	-	-	Restricted	Restricted	-	-	-	-	-	-	-	-
**ATBC**	77-90-7	≥ 10 000 to < 100 000	-	-	-	-	Restricted	Restricted	Restricted	-	-	-	-	-	-	-
**TOTM**	3319-31-1	≥ 10 000 to < 100 000	-	-	-		Restricted	Restricted	-	-	-	-	-	-	-	-

"-" Not banned or restricted

Table S2. Selected properties of plasticizers di(2-ethylhexyl) phthalate (DEHP) and di(2-propylheptyl) phthalate (DPHP).

	DEHP	DPHP
Chemical stucture	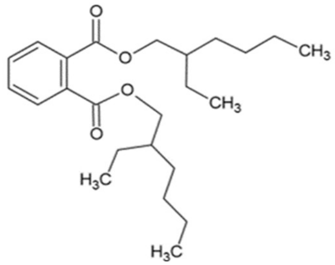	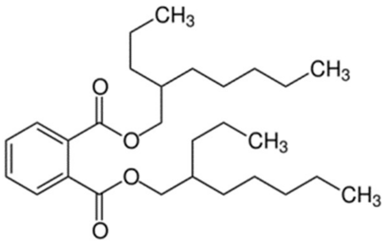
Molecular weight [g/mol]	391	447
Physcial state	Oily liquid	Oily liquid
Density [g/cm^3]	0.98	0.96
Water solubility [µg/L]	3	<0.1
Henry's law constant [Pa m^3/mol]	4.4	3.7
Log K_{OW}	7.52	10.4
Log K_{OC}	5.4	6.8

Table S3. Total number of independent experiments and replicates (data) for each toxicity endpoint in *Daphnia magna* assays (F0, F1 generation). Samples are non-spiked control (natural water), solvent (ethyl acetate) control and di(2-ethylhexyl) phthalate (DEHP) and di(2-propylheptyl) phthalate (DPHP) exposures. Exposure concentrations were 25 or 50 mg phthalate/kg sediment.

	Toxicity Endpoint	Sample	Independent experiments	Total data for endpoint
F0	Body length	non-spiked control	5	48
		solvent control	5	34
		DEHP25	5	39
		DEHP50	5	37
		DPHP25	5	29
		DPHP50	5	42
F1	Body length	non-spiked control	4	29
		solvent control	4	25
		DEHP25	4	21
		DEHP50	4	20
		DPHP25	4	24
		DPHP50	4	18
F0	Offspring/female	non-spiked control	2	48
		solvent control	2	34
		DEHP25	2	39
		DEHP50	2	37
		DPHP25	2	29
		DPHP50	2	42
F1	Offspring/female	non-spiked control	2	18
		solvent control	2	17
		DEHP25	2	18
		DEHP50	2	20
		DPHP25	2	24
		DPHP50	2	15
F0	Number of broods	non-spiked control	3	48
		solvent control	3	34
		DEHP25	3	39
		DEHP50	3	35
		DPHP25	3	27
		DPHP50	3	43
F1	Number of broods	non-spiked control	2	18
		solvent control	2	17
		DEHP25	2	18
		DEHP50	2	20
		DPHP25	2	24
		DPHP50	2	15
F0	Days to 1st brood	non-spiked control	5	50
		solvent control	5	34
		DEHP25	5	39
		DEHP50	5	37
		DPHP25	5	29
		DPHP50	5	42
F1	Days to 1st brood	non-spiked control	2	18
		solvent control	2	17
		DEHP25	2	18
		DEHP50	2	20
		DPHP25	2	25
		DPHP50	2	15

Table S4. Total number of independent experiments and data for each toxicity endpoint in *Porcellio scaber* and *Tenebrio molitor* assays. Samples are non-spiked control (soil or bran), solvent (acetone) control and di(2-ethylhexyl) phthalate (DEHP) and di(2-propylheptyl) phthalate (DPHP) exposures.

DEHP

Species	Toxicity Endpoint	Sample	Test jars	Total animals exposed	Total data for endpoint
Porcellio scaber	HC levels	non-spiked control	5	25	16
		solvent control	5	25	12
		5	5	25	10
		50	5	25	10
		200	5	25	10
		1000	5	25	10
	GC levels	non-spiked control	5	25	16
		solvent control	5	25	12
		5	5	25	10
		50	5	25	10
		100	5	25	10
	SGC levels	non-spiked control	5	25	16
		solvent control	5	25	12
		5	5	25	10
		50	5	25	10
		100	5	25	10
	THC levels	non-spiked control	5	25	16
		solvent control	5	25	12
		5	5	25	10
		50	5	25	10
		100	5	25	10
	AChE activity	non-spiked control	5	25	18
		solvent control	5	25	18
		5	5	25	15
		50	5	25	15
		100	5	25	14
	GST activity	non-spiked control	5	25	18
		solvent control	5	25	18
		5	5	25	15
		50	5	25	15
		100	5	25	14
	ETS activity	non-spiked control	5	25	18
		solvent control	5	25	18
		5	5	25	15
		50	5	25	15
		100	5	25	14
Tenebrio molitor	Larvae moult	non-spiked control	9	45	9
		solvent control	9	45	9
		5	9	45	9
		50	9	45	9
		100	9	45	9
		500	9	45	9
		1000	6	30	6
	Larvae growth	non-spiked control	9	45	9
		solvent control	9	45	9
		5	9	45	9
		50	9	45	9
		500	9	45	9
		1000	6	30	6
	Pupae emergence	non-spiked control	9	45	9
		solvent control	9	45	9
		5	9	45	9
		50	9	45	9
		500	9	45	9
		1000	6	30	6
	Adult emergence	non-spiked control	9	45	9
		solvent control	9	45	9
		5	9	45	9
		50	9	45	9
		500	9	45	9
		1000	6	30	6

DPHP

Species	Toxicity Endpoint	Sample	Test jars	Total animals exposed	Total data for endpoint
Porcellio scaber	HC levels	non-spiked control	5	25	10
		solvent control	5	25	10
		5	5	25	10
		50	5	25	10
		200	5	25	10
		1000	5	25	10
	GC levels	non-spiked control	5	25	10
		solvent control	5	25	10
		5	5	25	10
		50	5	25	10
		200	5	25	10
		1000	5	25	10
	SGC levels	non-spiked control	5	25	10
		solvent control	5	25	10
		5	5	25	10
		50	5	25	10
		200	5	25	10
		1000	5	25	10
	THC levels	non-spiked control	5	25	10
		solvent control	5	25	10
		5	5	25	10
		50	5	25	10
		200	5	25	10
		1000	5	25	10
	AChE activity	non-spiked control	5	25	10
		solvent control	5	25	10
		5	5	25	10
		50	5	25	10
		200	5	25	10
		1000	5	25	10
	GST activity	non-spiked control	5	25	10
		solvent control	5	25	10
		5	5	25	10
		50	5	25	10
		200	5	25	10
		1000	5	25	10
	ETS activity	non-spiked control	5	25	10
		solvent control	5	25	10
		5	5	25	10
		50	5	25	10
		200	5	25	10
		1000	5	25	10
Tenebrio molitor	Larvae moult	non-spiked control	6	30	6
		solvent control	6	30	6
		5	6	30	6
		50	6	30	6
		500	6	30	6
		1000	6	30	6
	Larvae growth	non-spiked control	6	30	6
		solvent control	6	30	6
		5	6	30	6
		50	6	30	6
		500	6	30	6
		1000	6	30	6
	Pupae emergence	non-spiked control	6	30	6
		solvent control	6	30	6
		5	6	30	6
		50	6	30	6
		500	6	30	6
		1000	6	30	6
	Adult emergence	non-spiked control	6	30	6
		solvent control	6	30	6
		5	6	30	6
		50	6	30	6
		500	6	30	6
		1000	6	30	6

Table S5. Comparison of organismal size and reproduction endpoints of *Daphnia magna* in F0 and F1 generations.

	Parental *D. magna* size		Days to 1st brood		Number of broods		Offspring/female	
	F0	F1	F0	F1	F0	F1	F0	F1
control	**3.63±0.17**	**3.46±0.23**	9.16±0.82	9.16±1.98	3.96±0.29	3.78±0.73	61.7±8.97	61.9±17.5
solvent	**3.55±0.20**	**3.38±0.19**	**9.32±0.81**	**8.94±1.89**	**3.79±0.41**	**4.18±0.73**	55.5±15.9	62.8±20.6
DEHP								
25 mg/kg	**3.47±0.19**	**3.17±0.30**	9.77±1.25	9.78±1.26	3.82±0.51	3.72±0.57	53.2±13.4	48.2±15.6
50 mg/kg	3.42±0.19	3.41±0.18	**10.1±1.65**	**8.95±0.51**	3.86±0.43	3.90±0.64	54.1±15.1	59.7±11.1
DPHP								
25 mg/kg	**3.53±0.15**	**3.37±0.16**	9.41±0.78	9.20±0.87	4.00±0.00	4.04±0.46	59.5±9.46	54.5±10.7
50 mg/kg	**3.45±0.20**	**3.32±0.14**	10.2±1.23	10.7±1.10	3.93±0.26	3.67±0.49	54.4±13.7	55.2±10.6

Solvent – ethyl acetate; DEHP- di(2-ethylhexyl) phthalate; DPHP- di(2-propylheptyl) phthalate. The F0-F1 data that are different ($p<0.05$) from each other in pairwise comparison are in bold font. Data are presented as AVG±SD (n=2-5).

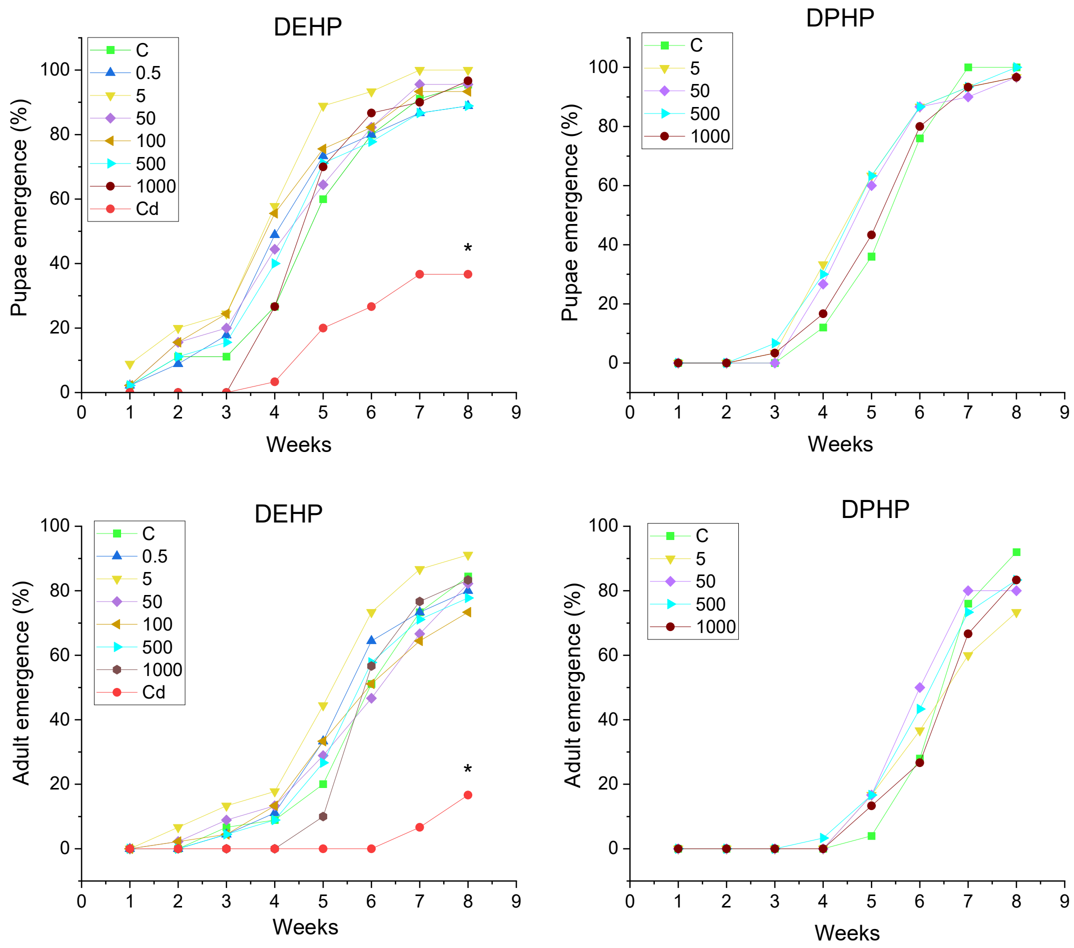

Figure S1. Emergence of *Tenebrio molitor* larvae and adults during the 8-week exposure to di(2-ethylhexyl) phthalate (DEHP) and di(2-propylheptyl) phthalate (DPHP). The concentration of the positive control chemical Cd was 5000 mg Cd/kg food (bran). Concentrations of DEHP or DPHP are in mg/kg food. C-control. The asterisks indicate a difference from solvent (acetone) control (*$p<0.05$; Kruskal-Wallis ANOVA, Dunn's test).

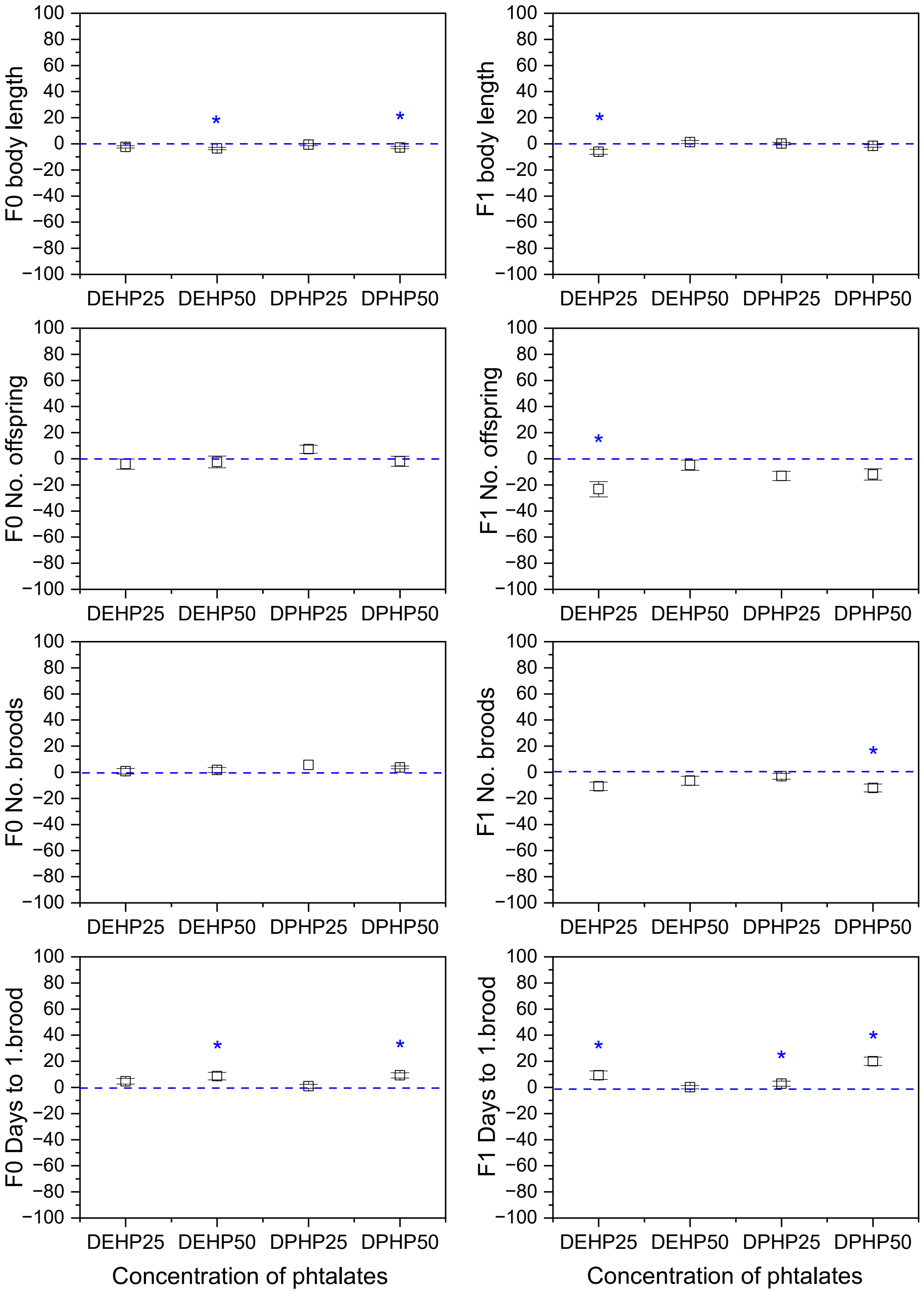

Figure S2. Control-normalised plots for *Daphnia magna*. Changes (%) in comparison to solvent control (AVG ± SE) are shown. Impact of di(2-ethylhexyl) phthalate (DEHP) and di(2-propylheptyl) phthalate (DPHP) on the size (body length) and reproduction (days to first brood, number of broods, offspring/female) of *Daphnia magna* in F0 and F1 generation is shown. Exposure concentrations were 25 or 50 mg phthalate/kg sediment. The asterisks indicate difference from the solvent (ethyl acetate) control. Blue line shows control value.

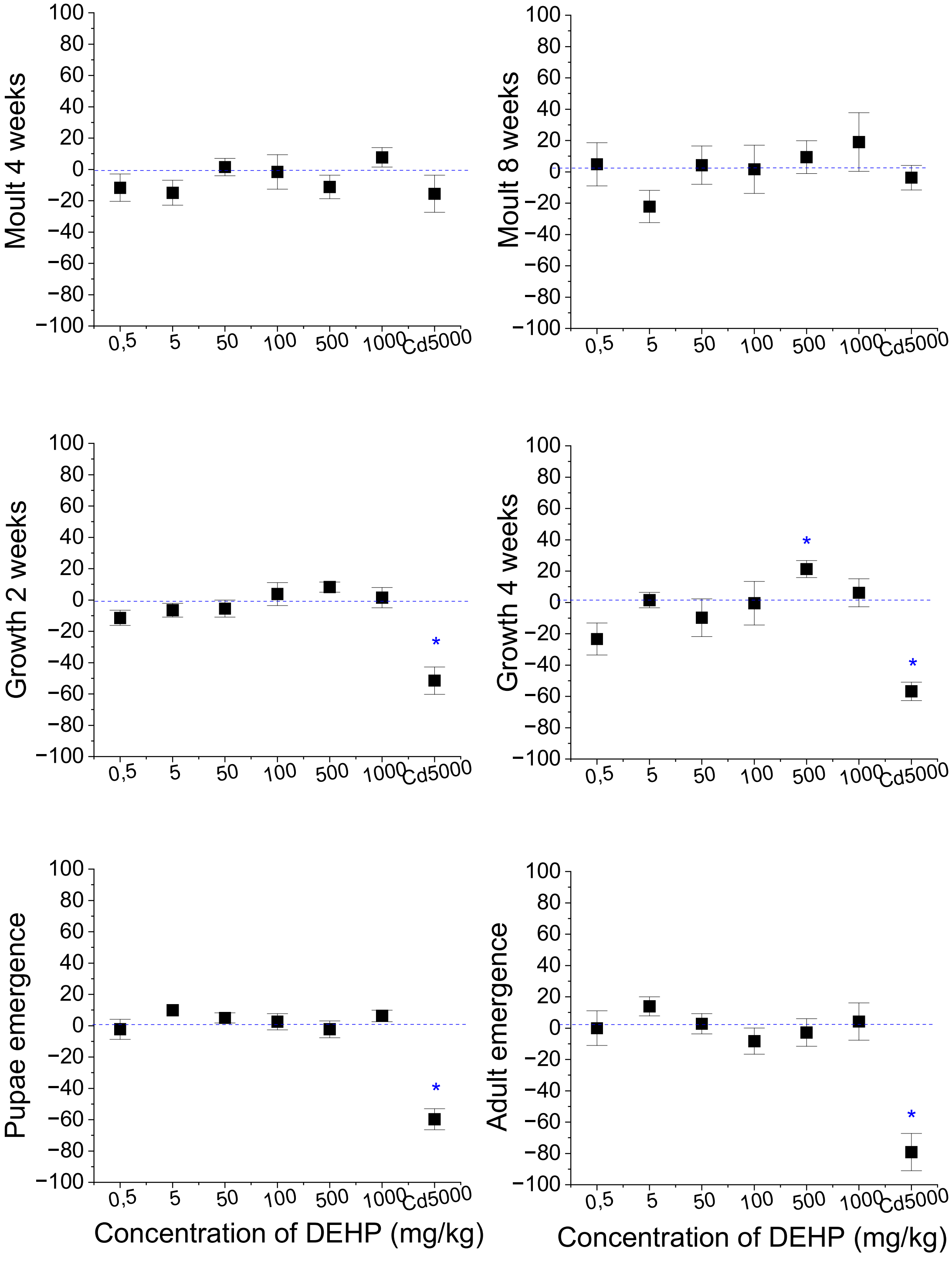

Figure S3. Control-normalised plots for *Tenebrio molitor* exposed to di(2-ethylhexyl) phthalate (DEHP). Changes (%) in comparison to solvent control (AVG ± SE) are shown. The asterisks indicate difference from the solvent (ethyl acetate) control. The concentration of the positive control chemical Cd was 5000 mg Cd/kg food (bran). Blue line shows control value.

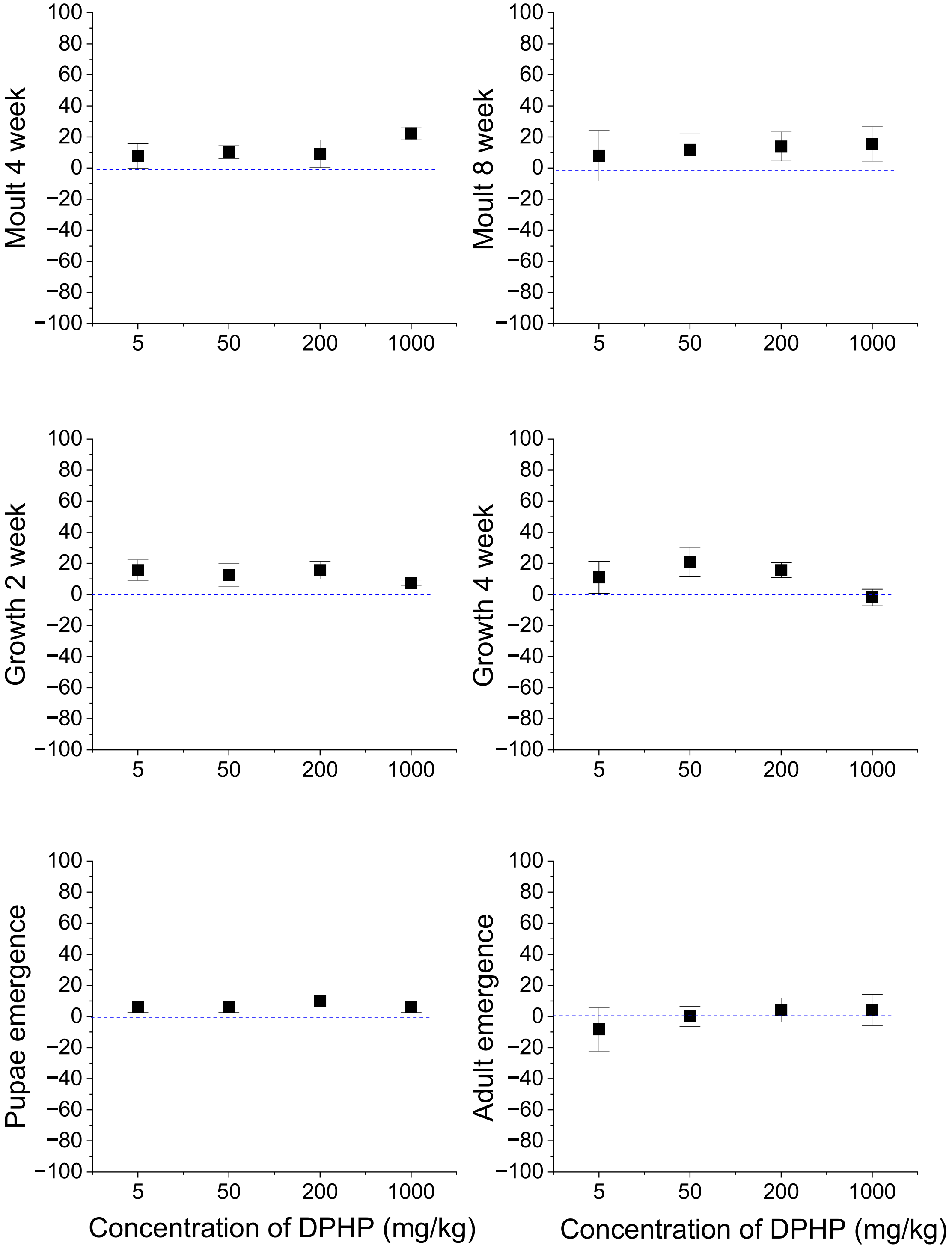

Figure S4. Control-normalised plots for *Tenebrio molitor* exposed to di(2-propylheptyl) phthalate (DPHP). Changes (%) in comparison to solvent control (AVG ± SE) are shown. The asterisks indicate difference from the solvent (ethyl acetate) control. The concentration of the positive control chemical Cd was 5000 mg Cd/kg food (bran). Blue line shows control value.

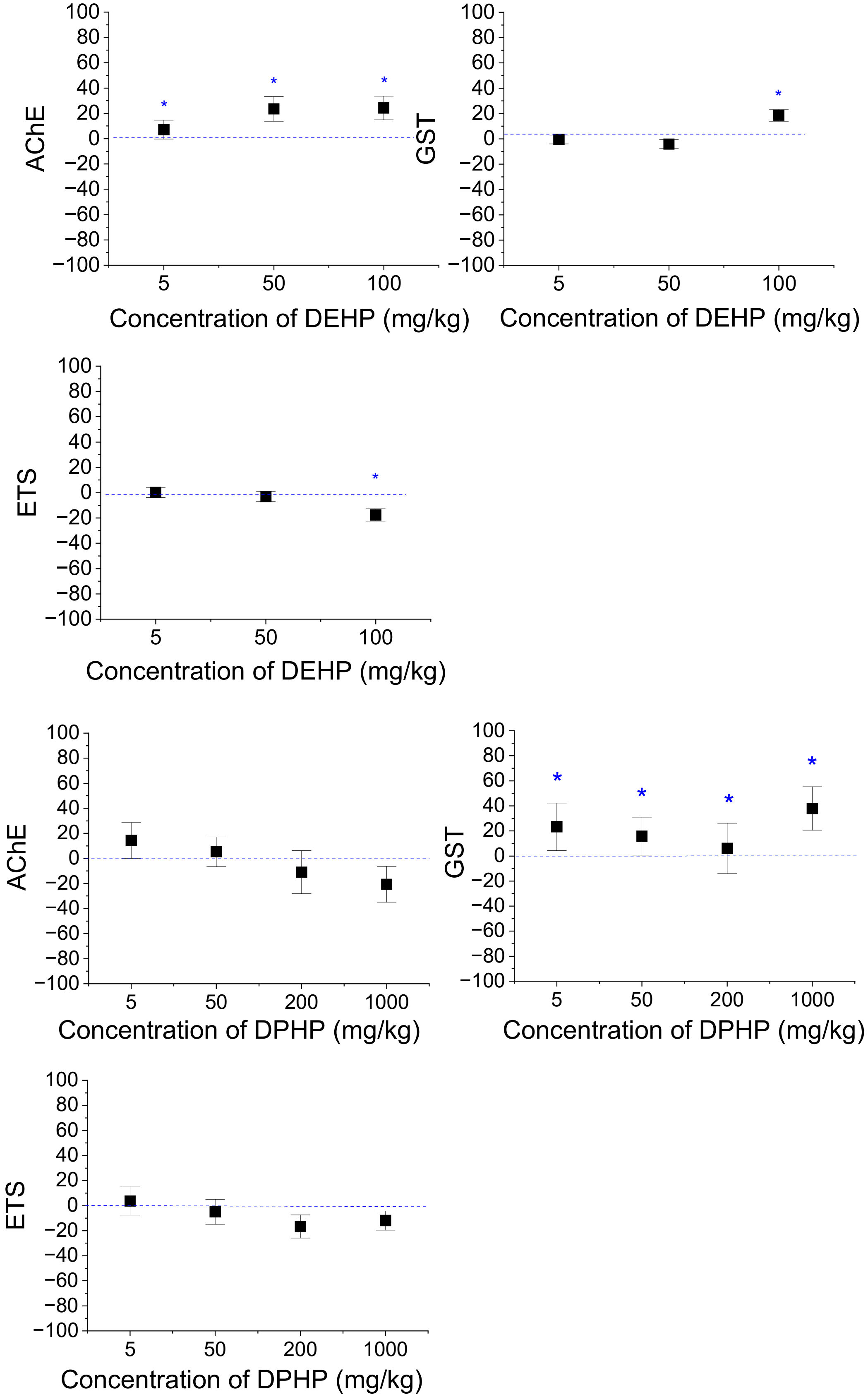

Figure S5. Control-normalised plots for *Porcellio scaber* exposed to di(2-ethylhexyl) phthalate (DEHP) and di(2-propylheptyl) phthalate (DPHP). Activities of acetylcholinesterase (AChE), glutathione S-transferase (GST) and electron transfer system activity (ETS) are shown. Changes (%) in comparison to solvent control (AVG ± SE) are shown.

References

1. Ellman, G. L.; Courtney, K. D.; Andres, V. Jr.; Featherstone, R. M. A New and Rapid Colorimetric Determination of Acetylcholinesterase Activity. Biochem. Pharmacol. 1961, 7, 88-95, DOI: 10.1016/0006-2952(61)90145-9

2. Habig, W. H.; Pabst, M. J.; Jakoby, W. B. Glutathione S-transferases. The First Enzymatic Step in Mercapturic Acid Formation. J. Biol. Chem. 1974, 249, 7130–7139. https://doi.org/10.1016/S0021-9258(19)42083-8

3. de Coen, W. M.; Janssen, C. R. The Use of Biomarkers in *Daphnia magna* Toxicity Testing. IV. Cellular Energy Allocation: A New Methodology to Assess the Energy Budget of Toxicant-Stressed *Daphnia* Populations. J. Aquat. Ecosyst. Stress Recovery 6, 1997, 43-55. https://doi.org/10.1023/A:1008228517955
